# Impact of human adipose tissue-derived stem cells on dermatofibrosarcoma protuberans cells in an indirect co-culture: an in vitro study

**DOI:** 10.1186/s13287-021-02512-5

**Published:** 2021-08-06

**Authors:** Zhaoqi Yuan, Zhu Zhu, Fangxing Zhu, Feixue Ding, Yinmin Wang, Xiuxia Wang, Xusong Luo, Jun Yang, Fei Liu, Di Sun

**Affiliations:** 1 Department of Plastic and Reconstructive Surgery, Shanghai Ninth People’s Hospital, Shanghai Jiao Tong University School of Medicine, Shanghai, 200011 China; 2 Shanghai Key Lab of Tissue Engineering, Shanghai Ninth People’s Hospital, Shanghai Jiao Tong University School of Medicine, Shanghai, 200011 China; 3 Department of Oral and Cranio-maxillofacial Surgery, Shanghai Ninth People’s Hospital, College of Stomatology, Shanghai Jiaotong University School of Medicine, Shanghai, 200011 China

**Keywords:** Dermatofibrosarcoma protuberans (DFSP), Adipose tissue-derived stem cells (ADSCs), Skin therapy, Regenerative medicine

## Abstract

**Background:**

Autologous adipose tissue transfer may be performed for aesthetic needs following the resection of dermatofibrosarcoma protuberans (DFSP), the most common cutaneous soft tissue sarcoma, excluding Kaposi sarcoma. The regenerative effectiveness of cell-assisted lipotransfer is dependent on the presence of adipose tissue-derived stem cells (ADSCs). This is the first study to evaluate the potential oncological risks as ADSCs could unintentionally be sited within the proximity of the tumor microenvironment of DFSP cells.

**Methods:**

Primary DFSP cells were indirectly co-cultured with ADSCs in a conditioned medium or in a Transwell system. The impact was analyzed by assessing proliferation, migration, invasion, angiogenesis, and tumor-associated genes and proteins. Results of these assays were compared between co-culture and mono-culture conditions.

**Results:**

Our experimental results showed that ADSCs were able to promote proliferation, migration, invasion, and angiogenesis of DFSP cells; this was accompanied by a significant increase in the expression levels of beta-type platelet-derived growth factor receptor, collagen type I alpha 1 chain, vascular endothelial growth factor, hepatocyte growth factor, and basic fibroblast growth factor.

**Conclusions:**

The current report clearly demonstrates that ADSCs can enhance different malignant properties of DFSP cells in vitro, which should not be neglected when considering the clinical use of human ADSCs and its related derivatives in skin regenerative therapies.

## Background

Human mesenchymal stem/stromal cells (hMSCs) are a heterogeneous ensemble of cells with fibroblast-like morphology and can proliferate and form colonies in vitro. Additionally, hMSCs are capable of undergoing multilineage differentiation. Owing to the beneficial immunomodulatory and regenerative properties of hMSCs, these cells have received significant attention as potential agents for therapies [1]. Adipose tissue-derived stem cells (ADSCs), an abundant and readily available subset of hMSCs, can be largely extracted from subcutaneous human adipose tissue; thus, they are one of the most suitable cell sources for stem cell-based therapies [2]. ADSCs have tremendous plasticity with tri-lineage differentiation potential; these cells can differentiate into osteocytes, chondrocytes, and adipocytes [3]. ADSCs can affect cells in their microenvironment through the paracrine secretion of proteins [4]. Owing to their self-renewal, unlimited proliferative, proangiogenic, and immunomodulatory properties, ADSCs have been used as attractive adjuncts in the form of cell-assisted lipotransfer to improve wound healing, angiogenesis, tissue engineering, and soft tissue augmentation after reconstructive surgery [5]. Recently, it has been reported that ADSCs loaded with biomaterials as antitumor drug carriers selectively target solid tumors during thermo-/chemotherapy. This can improve the typical drug delivery methods, correlating with magnetic resonance imaging tracking for diagnostic applications [6, 7].

Interestingly, ADSCs have also been shown to exhibit duality. These cells not only greatly promote cell regeneration but also facilitate the progression of tumors [1, 8]. ADSCs have been reported to be actively recruited into the tumor nidus and surrounding inflammatory microenvironment by cancer cells, thus increasing tumor vascularity [9]. Moreover, ADSCs have been suggested to differentiate into cancer-associated fibroblasts (CAFs), which form an essential part of the tumor stroma [10]. Therefore, they serve as an important promoter of tumor growth, invasion, and metastasis through the secretion of various cytokines and proteases [11, 12]. Additionally, ADSCs are similar to CAFs in their cancer-promoting properties; therefore, ADSCs interact within the tumor microenvironment (TME), promoting cancer cell proliferation, viability, invasiveness, and chemoresistance [3]. Consequently, there have been concerns regarding the oncological safety of using ADSCs in cell-based regenerative therapy for reconstruction after cancer surgery [8]. Although studies focusing on ADSCs and tumors have been gaining attention, none of these studies have examined the impact of ADSCs on dermatofibrosarcoma protuberans (DFSP).

DFSP is a rare, low-grade, soft tissue sarcoma; it is the most common type of cutaneous soft tissue sarcoma, excluding Kaposi sarcoma [13]. Problems such as initial misdiagnoses, prolonged time to accurate diagnosis, and large tumor size at the time of diagnosis are common because of the lack of specific DFSP characteristics [14]. In addition, with highly irregular shapes [15], these tumor cells often infiltrate the dermis and spread into the underlying subcutaneous tissue, which results in an incomplete resection and a high recurrence rate of DFSP [16]. Hence, to achieve clear surgical margins, extended excision is necessary, which causes large defects in the skin and soft tissue. It is challenging for plastic and reconstructive surgeons to repair thesis defects, including morphological alteration and function loss. Most recently, some studies have mentioned fat grafting at the surgical site of malignant neoplasms of mesenchymal origin, although these studies have cautioned against its potential safety issues [17]. Evidence of the oncological safety of fat grafting after cancer surgery is based primarily on clinical studies in breast cancer and is limited by possible bias. The biology of sarcoma is relatively different from that of breast carcinoma. The unclear and diverse etiopathogenesis of sarcomas and high risk of local recurrence combined with the variable signaling of the fat microenvironment, including ADSCs and related growth factors, should be investigated more extensively [17].

Therefore, considering the growing use of fat, stem cell-enriched, and isolated ADSCs in defects of the skin and soft tissues after radical tumor resection, it is important to identify the oncological safety of possible interactions between co-localized ADSCs and DFSP cells. Unfortunately, there is no report on this issue. This is the first study to co-culture primary DFSP cells with ADSCs. Subsequently, we quantified the changes in proliferation, migration, invasion, and angiogenesis and compared the results with those of DFSP cell mono-cultures. In addition, we aimed to obtain further insight into the interactions from the perspective of genes and proteins. If ADSCs exert a promoting effect on DFSP cells, it may alert plastic surgeons about potential safety issues as these ADSCs are injected into a surgical site where possibly residual or dormant DFSP cells can survive and develop.

## Methods

### Adipose tissue-derived stem cell (ADSC) isolation, cultivation, and identification

Human adipose tissue samples were obtained by liposuction of the abdominal wall from three different donors. After rinsing three times with phosphate-buffered saline (PBS), the samples were digested with 0.1% (w/v) collagenase IV (NB4; Serva, Heidelberg, Germany) for 2 h. Cells were concentrated by centrifugation at 1500 rpm at 37°C for 5 min to obtain ADSCs. ADSCs were cultured in low-glucose Dulbecco’s modified Eagle’s medium (DMEM; Hyclone, Logan, UT, USA) supplemented with 10% fetal bovine serum (FBS; ScienCell Research Laboratories, Inc., San Diego, CA, USA), 100 U/mL penicillin, and 100 μg/mL streptomycin (Gibco; Thermo Fisher Scientific, Inc., Waltham, MA, USA) at 37°C in 5% CO_2_ until they reached 80–90% confluence. Thereafter, they were dissociated with 0.05% trypsin-ethylenediaminetetraacetic acid and passaged. The cells of passages 2–6 were combined and used for further characterization and in vitro differentiation [18].

### ADSC identification

The capacity of ADSCs to differentiate into osteoblasts, chondrocytes, and adipocytes was assessed as described [19–21].

#### Osteogenic differentiation assay

Human ADSCs (2×10^4^ cells/cm^2^) were seeded in six-well plates that were pre-coated with a 0.1% gelatin solution and then cultured in DMEM containing 10% FBS, 1% antibiotic/antimycotic, 0.01 μM 1,25-dihydroxyvitamin D3, 50 μM ascorbate-2-phosphate, and 10 mM β-glycerophosphate (HUXMD-90021, Cyagen Bioscience, Inc., Santa Clara, CA, USA). The medium was changed every 3 days. After 28 days of culture at 37°C under 5% CO_2_, the cells were washed twice with PBS, fixed in 4% paraformaldehyde for 30 min, and stained with 0.3% Alizarin red for 5 min. After two washes with PBS, the cells were observed and photographed under a phase-contrast inverted microscope (Olympus, Tokyo, Japan).

#### Chondrogenic differentiation assay

Human ADSCs (4×10^5^ cells) were seeded in 15 mL centrifuge tubes filled with DMEM containing 10% FBS, 1% antibiotic/antimycotic, 6.25 μg/mL insulin, 10 ng/mL transforming growth factor-beta 1, and 50 nM ascorbate-2-phosphate (HUXMD-90041, Cyagen Bioscience, Inc.). The medium was changed every 3 days. After 28 days of culture at 37°C under 5% CO_2_, the cartilage balls were formalin-fixed, paraffin-embedded, sectioned, and stained with Alcian blue. The sections were observed and photographed under a light microscope (Leica Microsystems GmbH, Wetzlar, Germany).

#### Adipogenic differentiation assay

Human ADSCs (2×10^4^ cells/cm^2^) were seeded in six-well plates in DMEM containing 10% FBS, 1% antibiotic/antimycotic solution, 0.5 mM isobutyl-methylxanthine, 1 μM dexamethasone, 10 μM insulin, and 200 μM indomethacin (Cyagen Bioscience, Inc., HUXMD-90031). The medium was changed every 3 days. After 28 days of culture at 37°C under 5% CO_2_, the cells were washed twice with PBS, fixed in 4% paraformaldehyde for 30 min, and stained with 0.3% oil red O solution for 30 min. After two washes with PBS, the cells were observed and photographed under a phase-contrast inverted microscope (Olympus).

### Flow cytometric assay

Flow cytometric analysis was used to identify the markers of ADSCs according to a published paper [22]. Briefly, hADSCs were harvested and washed thrice with PBS. The cell suspension was incubated with fluorescein isothiocyanate-conjugated antibodies against CD29, CD31, CD45, and CD90 (Santa Cruz Biotechnology, Inc., Santa Cruz, CA, USA) and phycoerythrin-conjugated antibodies against CD105 and CD44 (Santa Cruz Biotechnology, Inc.) at 37°C for 30 min in the dark, washed, and resuspended in PBS and subjected to flow cytometry (BD Biosciences, San Jose, CA, USA).

### Dermatofibrosarcoma protuberans (DFSP) cell isolation and cultivation

Three DFSP samples from one man and two women were obtained after excision from the corresponding sites (Table 1). Samples were soaked in chloromycetin for 30 min, cut into as small pieces as possible, and then digested with 0.1% collagenase IV for 30 min at 37°C. After centrifugation, cells were suspended in high-glucose DMEM (Hyclone, Logan, UT, USA) with 10% FBS, 100 U/mL penicillin, and 100 mg/mL streptomycin at 37°C in 5% CO_2_. Cells at passages 2–6 were used in this experiment [23, 24].

**Table 1:** Clinical information of patients with dermatofibrosarcoma protuberans

Sample	Sex	Age	Location
1	1	30–39	Anterior chest
2	2	40–49	Abdomen
3	1	40–49	Left clavicle

### DFSP-ADSC co-cultures

#### Indirect co-culture by a conditioned medium

To prepare a human ADSC conditioned medium (CM) [25], 2×10^5^ ADSCs were seeded onto a six-well cell culture plate with DMEM/F12 (Hyclone, Logan, UT, USA) medium containing 10% FBS overnight, and the culture medium was replaced with DMEM/F12 serum-free (SF) and conditioned for 24 h. ADSC-CM was harvested, filtered through 0.22-μm filters (Jet Bio-Filtration, Guangzhou, China), and stored at −80°C until use.

#### Indirect co-culture by a Transwell system

**Fig. 1:**
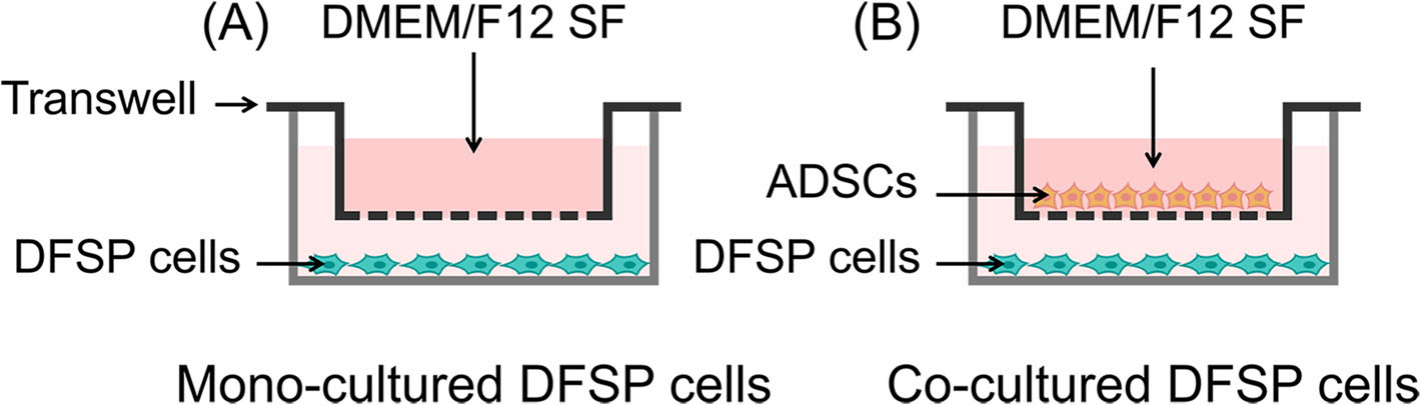
Model of DFSP cells co-cultured with ADSCs by Transwell. **A** DFSP cells cultured alone and **B** DFSP cells co-cultured with ADSCs in DMEM/F12 SF. ADSCs, adipose tissue-derived stem cells; DFSP, dermatofibrosarcoma protuberans; DMEM, Dulbecco’s modified Eagle’s medium; SF, serum-free

Co-culture of DFSP cells and ADSCs was performed using a Transwell system (Fig. 1) [12, 25, 26]. First, 2×10^5^ ADSCs were seeded onto a polyester membrane Transwell-clear insert (pore size 0.4 μm, Corning Incorporated, Corning, NY, USA). Next, DFSP cells were seeded onto the bottom of a six-well cell culture plate with the same cell density in DMEM/F12 medium containing 10% FBS overnight. Subsequently, the medium was replaced with fresh DMEM/F12 SF to eliminate non-adherent cells. At the required time point, DFSP cells or supernatants (co-cultured DFSP/ADSC-CM) were collected and stored at −80°C until use. Isolated DFSP cells seeded on six-well culture plates served as controls and were treated as the co-cultured cells.

### Proliferation assays

The ability of ADSC-CM to induce DFSP cell proliferation was assessed using a Cell Counting Kit-8 (CCK-8) (Dojindo Laboratories, Kumamoto, Japan) according to the manufacturer’s protocols [23, 27]. Briefly, DFSP cells (2×10^3^/well) were seeded in 96-well plates. After 24 h, the DFSP cells were treated with ADSC-CM (supplemented with 1% FBS) and DMEM/F12 (supplemented with 1% FBS) as a control. The medium was replaced every 48 h. After 1, 3, 5, and 7 days, the cells were washed three times with PBS, and 100 μL of fresh culture medium with 10 μL of CCK-8 reagent was added to each well and incubated at 37°C for 2 h. The absorbance of each sample, which was proportional to the number of viable cells, was measured at a wavelength of 450 nm using a microplate reader (Thermo Fisher Scientific, Waltham, MA, USA). Each group was prepared in triplicates. The experiment was repeated three times.

### Cell migration assays

#### Scratch wound healing

DFSP cells (2×10^5^/well) were seeded in six-well culture plates and grown in a complete medium until they reached 80% confluence. Subsequently, monolayers were scratched using a 200-μL sterile plastic pipette tip (PipetTipFinder, LLC, Knoxville, TN, USA), which was placed perpendicular to the bottom of the dish as previously described and then washed three times with PBS [28]. DFSP cells were then treated with ADSC-CM SF for 24 h. Positive controls were set up with DMEM/F12+10% FBS and negative controls with DMEM/F12 SF. Scratch wound closure was monitored using a phase-contrast inverted microscope (Olympus) at 0, 6, 12, and 24 h. It was then employed to measure the area between the opposite edges of the wound, which was semi-quantified with ImageJ software 1.46r (National Institutes of Health, USA). The migration rate was calculated using the following formula: migration rate (%)=(Gap0h-Gap24h)/Gap0h×100%. In each sample, five views were randomly photographed to obtain the mean, and the final mean rate plus standard deviation (mean±SD) was derived from the means of three cell samples [18].

#### Transwell assay

Cell migration [12, 25, 26] was also evaluated using a 24-well Transwell chamber (Corning, pore size 8.0 μm). For this purpose, once 80% confluence was achieved, DFSP cells were collected and seeded in the upper chamber of an 8.0-μm pore size insert (2×104 cells/well) with DMEM/F12 SF and allowed to migrate toward DMEM/F12+10% FBS (positive control), DMEM/F12 SF (negative control), or ADSCs (2×104 cells/well; experimental) present in the lower chamber. After incubation for 12 and 24 h, the non-migrating cells in the upper chamber were removed with a cotton swab, and the remaining cells were fixed in methanol for 30 min. Cells that migrated to the lower surface of the membrane were stained with 0.5% crystal violet, diluted for 5 min, gently washed three times in PBS, air-dried, and observed and photographed with a microscope (Olympus). Five fields were randomly selected for each assay. Quantification was performed by processing all obtained images using ImageJ software 1.46r (National Institutes of Health). The average number of migrating cells in five random fields was taken as the cell migration number of the group. All experiments were repeated three times.

### Cell invasion assay

The capacity of ADSC-CM to induce DFSP cell invasion was tested using a 24-well Transwell chamber (8-μm pore size insert) pre-coated with Matrigel matrix (Cat. No. 356234, Corning Incorporated) according to the manufacturer’s instructions [12, 25, 26]. Briefly, 100 μL of the diluted Matrigel matrix (800 μg/mL in SF medium) was carefully added to the center of each Transwell insert for invasion assays. The plate was incubated at 37°C for 1 h to allow the Matrigel to form a gel. The DFSP cells were counted and diluted to a density of 2×10^5^ cells/mL with DMEM/F12+10% FBS (positive control), DMEM/F12 SF (negative control), and ADSC-CM (experimental). Next, 100 μL of the cell suspension was seeded into the upper chamber of each Transwell. Thereafter, 800 μL of culture medium with 5% FBS was added to the lower chambers. The cells were cultured in a humidified incubator at 37°C with 5% CO_2_ for 36 h. The cells inside the Transwell inserts were gently removed using cotton swabs, and the cells on the lower surface of the membrane were stained with crystal violet for 5 min. The Transwell inserts were washed three times with PBS to remove unbound crystal violet and then air-dried. The invaded cells were observed and photographed under a microscope (Olympus). Five fields were randomly selected for each assay. Quantification was performed by processing all obtained images using ImageJ software 1.46r (National Institutes of Health). The average number of migrating cells in these five fields was taken as the cell invasion number of the group. All experiments were repeated three times.

### Angiogenic properties assay

To evaluate the effect of proteins secreted by isolated DFSP cells or ADSCs or both co-cultured cells on angiogenesis, SF CM of each condition was collected at 24 h of cell culture (processing with the abovementioned method). Human umbilical vein endothelial cells (HUVECs) were obtained from the Cell Bank of the Shanghai Institute of Cell Biology, Chinese Academy of Sciences (Shanghai, China). Wells of a 96-well plate were coated with Matrigel matrix, and 1×10^4^ HUVECs were seeded onto the matrix in each well. The ADSC-CM, DFSP-CM, and co-cultured DFSP/ADSC-CM were added and incubated for 4 h. Tube formation was visualized using bright-field microscopy. Quantification was performed by processing all obtained images using ImageJ software 1.46r (National Institutes of Health). The experiments were independently reproduced at least three times [12, 29, 30].

### Quantitative real-time polymerase chain reaction

After 24 h of co-culture (processing with the above method found in the “Indirect co-culture by a Transwell system” section), total RNA was extracted using EZ-press RNA Purification Kit (B0004D, EZBioscience, Roseville, USA) according to the manufacturer’s instructions [31–33]. RNA purity was evaluated by calculating the A260/A280 ratio, which should be between 1.8 and 2.0. The mRNA was reverse transcribed into cDNA with 4×Reverse Transcription Master Mix (A0010, EZBioscience). Subsequently, real-time quantitative real-time polymerase chain reaction (qRT-PCR) was performed using the cDNA as a template and 2×SYBR Green qPCR Master Mix (A0001, EZBioscience) according to the manufacturer’s instructions. Primers were synthesized by Sangon Biotech Co. (Shanghai, China). Target gene expression levels were normalized to glyceraldehyde 3-phosphate dehydrogenase and quantified using the comparative Ct method. The mean minimal cycle threshold value was calculated from triplicate reactions. The primers used for qRT-PCR analysis are listed in Table 2.

**Table 2:** Primers for RT-PCR

Gene	Forward primer (5'–3')	Reverse primer (5’–3’)
GAPDH	GGGAAGCTTGTCATCAATGGAA	AGAGATGATGACCCTTTTGGCTC
PDGFRB	AGCACCTTCGTTCTGACCTG	TATTCTCCCGTGTCTAGCCCA
COL1A1	GAGGGCCAAGACGAAGACATC	CAGATCACGTCATCGCACAAC
VEGF	AGGGCAGAATCATCACGAAGT	AGGGTCTCGATTGGATGGCA
HGF	GCTATCGGGGTAAAGACCTACA	CGTAGCGTACCTCTGGATTGC
bFGF	AGAAGAGCGACCCTCACATCA	CGGTTAGCACACACTCCTTTG

### Western blotting analysis

After 48 h of co-culture (processing with the above method found in the “Indirect co-culture by a Transwell system” section), total protein was extracted from the DFSP cells with radioimmunoprecipitation assay lysis buffer as described previously [34]. Supernatants were collected, and their total protein concentration was determined using the BCA Protein Assay Kit (Cat. No. P0010, Beyotime, Shanghai, China). Protein samples were completely denatured by boiling in bromophenol blue sample buffer for 5 min, separated by electrophoresis on 7.5% sodium dodecyl sulfate-polyacrylamide gel, and then transferred onto polyvinylidene difluoride membranes. Non-specific antibody binding was blocked with 5% non-fat dry milk in Tris-buffered saline containing 0.1% Tween 20 for 1 h at room temperature. The primary antibodies used were as follows: beta-type platelet-derived growth factor receptor (PDGFRB) (1:5000, Cat. No. ab32570, Abcam, Cambridge, MA, USA) and collagen type I alpha 1 chain (COL1A1) (1:1000, Cat. No. ab34710, Abcam). Immunoblotting was performed using specific primary antibodies and secondary antibodies conjugated to horseradish peroxidase. Protein blots were developed using an enhanced chemiluminescence method. β-actin (1:2000, Cat. No. ab8226, Abcam) was used as a loading control, and the results were analyzed as the detected β-actin ratio.

### Enzyme-linked immunosorbent assay

After 48 h of culturing, supernatants were collected from the DFSP-mono-culture and DFSP-ADSC-co-culture in Transwell (processing with the above method found in the “Indirect co-culture by a Transwell system” section) and stored at −80°C until use. The levels of vascular endothelial growth factor (VEGF), hepatocyte growth factor (HGF), and basic fibroblast growth factor (bFGF) were measured using an enzyme-linked immunosorbent assay (ELISA) kit (R&D Systems, Inc., Minneapolis, MN, USA) according to the manufacturer’s protocol [35, 36]. Standard curves were generated to calculate the cytokine levels. The experiments were independently repeated thrice.

### Statistical analyses

All quantitative results are presented as means±SDs. Statistical comparisons were performed using a Student’s *t* test in three independent experiments. GraphPad Prism version 7.0 software (GraphPad Inc., La Jolla, CA, USA) was used for data analysis. Statistical significance was set at *p*<0.05.

## Results

### ADSCs displayed multipotent differentiation and expressed stem cell markers

**Fig. 2:**
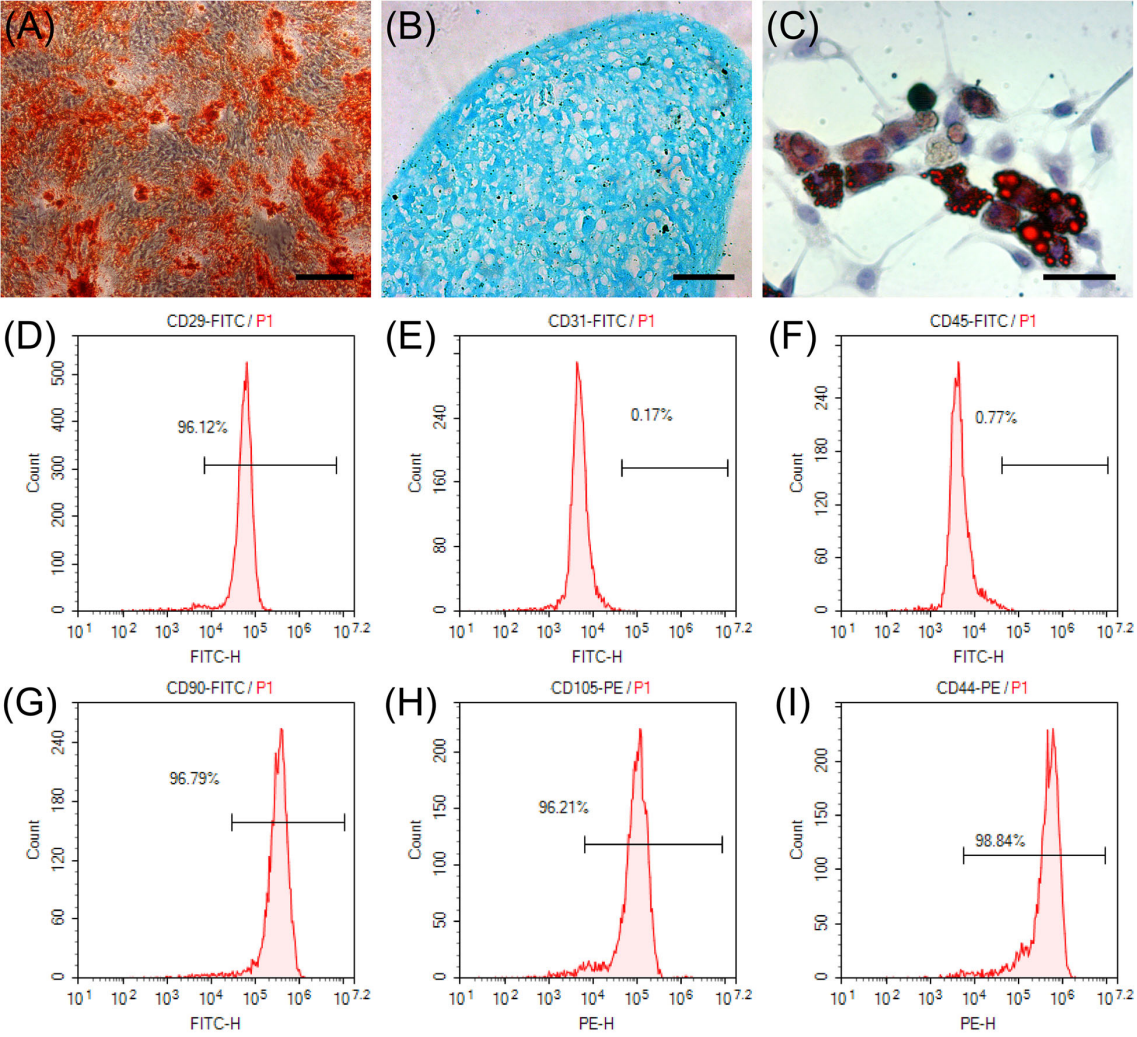
Characterization of human adipose-derived stem cells (ADSCs). The differentiation into **A** Alizarin Red-stained osteocytes (scale bar=100 μm), **B** Alcian blue-stained chondrocytes (scale bar=50 μm), and **C** oil red O-stained adipocytes (scale bar=50 μm) was induced. Flow cytometric analysis of ADSCs: ADSCs expressed **D** CD29+ (96.12%), **E** CD31+ (0.17%), **F** CD45+ (0.77%), **G** CD90+ (96.79%) marked with fluorescein isothiocyanate (FITC), **H** CD105+(96.21%), and **I** CD44+(98.84%) marked with phycoerythrin (PE)

The multipotency of ADSCs was examined by osteogenic, chondrogenic, and adipogenic differentiation assays. ADSCs were cultured and induced with osteogenic medium for 4 weeks and stained with Alizarin red to confirm the presence of calcium deposits (Fig. 2A). ADSCs were induced with chondrogenic medium for 4 weeks, and sections of cartilage balls were stained with Alcian blue (Fig. 2B). ADSCs were induced with adipogenic medium for 4 weeks and developed an adipogenic phenotype, which showed the presence of lipid droplets in the cells by oil red O staining (Fig. 2C). The ADSCs were characterized using mesenchymal stem cell surface markers as CD29+ (96.12%), CD31+ (0.17%), CD45+ (0.77%), CD90+ (96.79%), CD105+ (96.21%), and CD44+ (98.84%) (Fig. 2D–I) [22]. These results revealed that the ADSCs isolated from the human adipose tissue demonstrated typical ADSC characteristics.

### ADSC-CM promoted DFSP cell proliferation

**Fig. 3:**
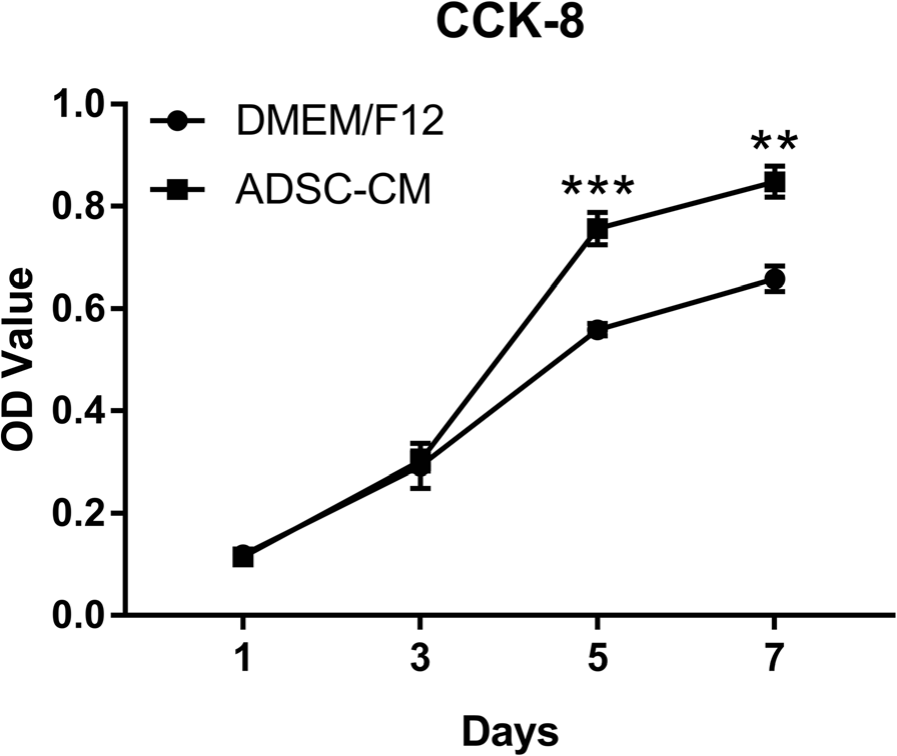
Effect of ADSC-CM on DFSP cell proliferation by CCK-8. CCK-8 assay was performed to measure proliferation rate in DFSP cells, which were treated with ADSC-CM or DMEM/F12 (control), at 1, 3, 5, and 7 days. ^**^p<0.01 and ^***^p<0.001 indicate significant differences between the two groups in three independent experiments. ADSCs, adipose tissue-derived stem cells; CCK-8, Cell Counting Kit-8; DFSP, dermatofibrosarcoma protuberans; DMEM, Dulbecco’s modified Eagle’s medium

The CCK-8 assay was used to evaluate the effect of ADSC-CM on DFSP cell proliferation. As shown in Fig. 3, ADSC-CM significantly promoted DFSP cell proliferation at days 5 and 7 compared with that in the control condition (*p*<0.05). There were no significant differences in cell proliferation rates between the experimental and control conditions during the first 3 days.

### ADSC-CM or ADSCs promoted DFSP cell migration

**Fig. 4:**
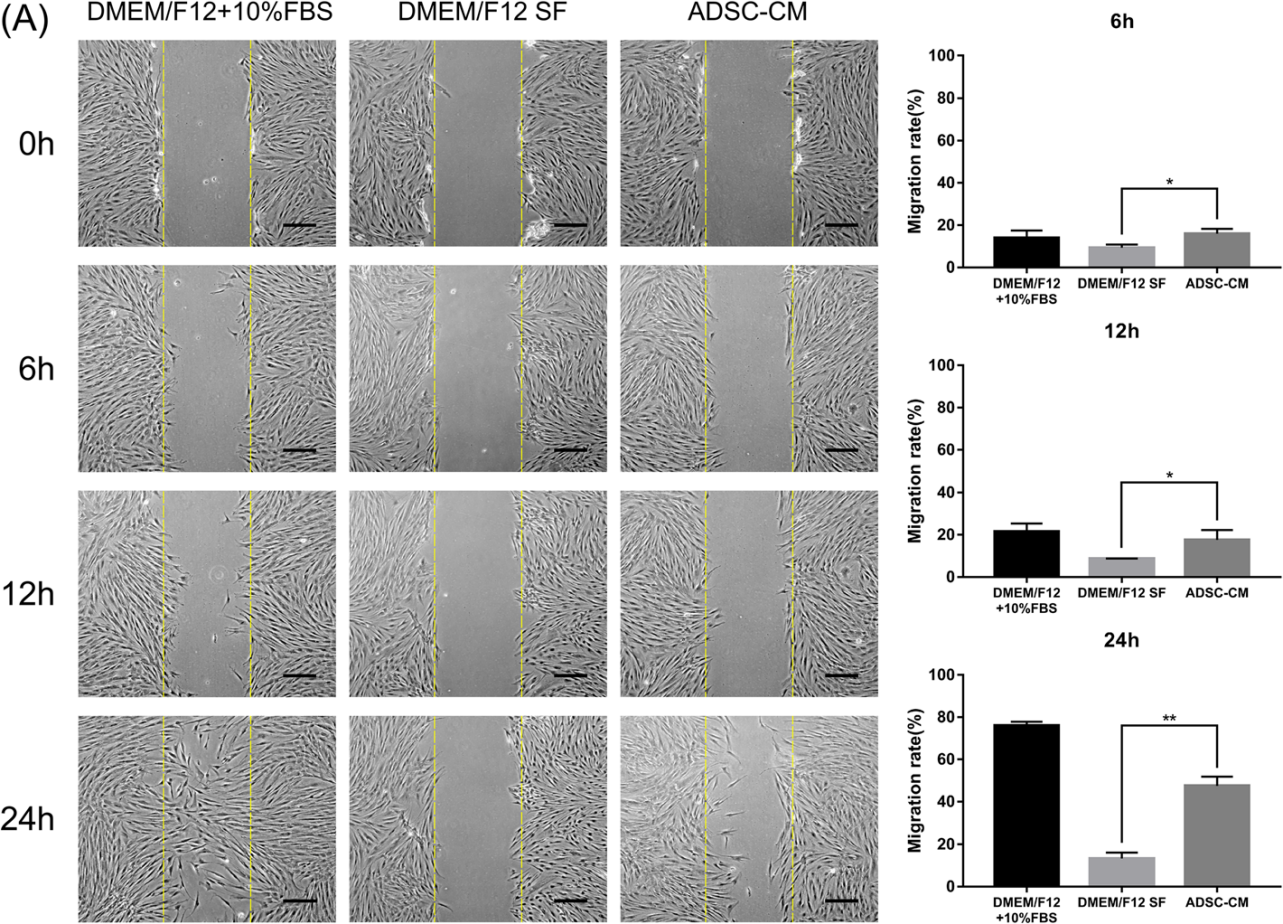
Effect of ADSC-CM on DFSP cell migration by scratch wound healing. The average initial wound width was measured and defined as 100%. **A** Inverted microscopic images of DFSP cell wound repair. DFSP cells were treated with ADSC-CM (experimental), DMEM/F12+10% FBS (positive controls), or DMEM/F12 SF (negative controls). Wound healing within the scrape line was recorded at 0, 6, 12, and 24 h. Yellow dashed lines indicate the margin of the scratch. Scale bars=75 μm. Semi-quantification of migration rate at **B** 6 h, **C** 12 h, and **D** 24 h post-wounding. The wound areas were quantified in five random low-power fields per well using an inverted microscope. ^*^p<0.05 and ^**^p<0.01 indicate significant differences between the ADSC-CM and negative control groups in three independent experiments. ADSCs, adipose tissue-derived stem cells; CM, conditioned medium; DFSP, dermatofibrosarcoma protuberans; DMEM, Dulbecco’s modified Eagle’s medium; SF, serum-free

We evaluated the ability of ADSCs to affect DFSP cell migration. First, as shown in Fig. 4A, the ability of ADSC-CM to affect DFSP cell migration was tested using a scratch wound model in which DFSP cells were treated with ADSC-CM (experimental), DMEM/F12+10% FBS (positive control), or DMEM/F12 SF (negative control). At 6 h, DFSP cells of the negative control (DMEM/F12 SF) group migrated to 9.3±1.5% of the scratched area, whereas the ADSC-CM-treated DFSP cells migrated to 15.9±2.3% of the area (*p*<0.05, Fig. 4B). At 12 h, the DFSP cells of the negative control group had migrated to 8.7±0.1% of the scratched area, whereas the ADSC-CM-treated DFSP cells migrated 17.5±4.7% of the area (*p*<0.05, Fig. 4C). At 24 h, the DFSP cells from the negative control group had migrated to 13.3±2.8% of the scratched area, whereas the ADSC-CM-treated DFSP cells had migrated 47.4±4.4% of the area (*p*<0.05, Fig. 4D). These results showed that treatment with ADSC-CM significantly promoted the migration of DFSP cells at 6, 12, and 24 h (1.71-, 2.01-, and 3.56-fold, respectively) compared with the negative control (DMEM/F12 SF).

**Fig. 5:**
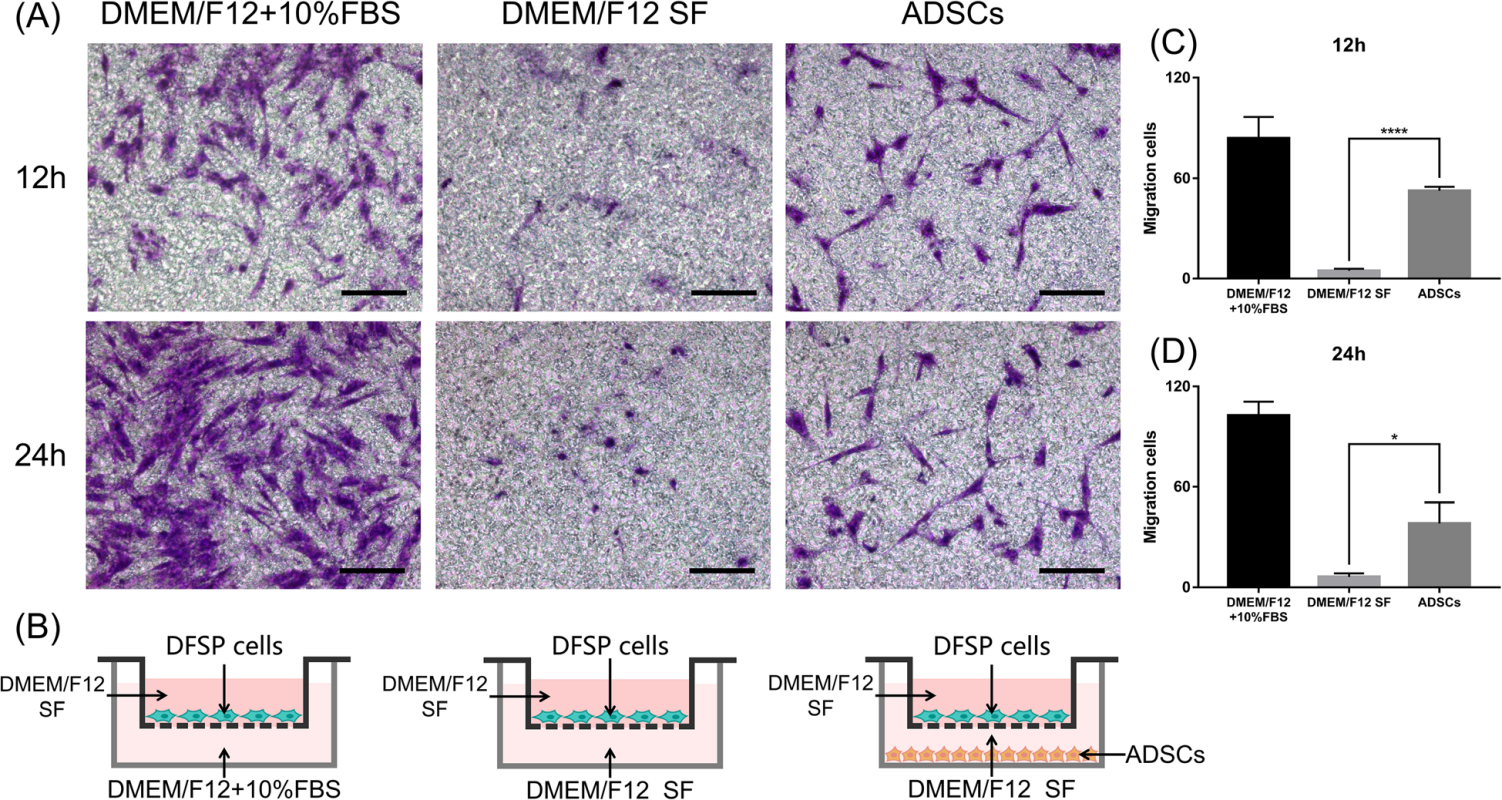
Effect of ADSCs on DFSP cell migration by a Transwell assay. **A** The migratory DFSP cells were visualized by staining cells with crystal violet. Scale bars=50 μm. **B** Illustrations of Transwell co-culture systems in different conditions: DFSP cells were seeded onto the upper chambers with DMEM/F12+10% FBS (positive control), DMEM/F12 SF (negative control), or ADSCs (experimental) added to the lower chambers; then, cell migration was determined at 12 and 24 h. The numbers of migrated cells at 12 h (**C**) and 24 h (**D**). Migrated cells were counted in five random low-power fields per chamber using an inverted microscope. ^*^p<0.05 and ^****^p<0.0001 indicate significant differences between co-culture with the ADSCs (experimental) and negative control groups in three independent experiments. ADSCs, adipose tissue-derived stem cells; DFSP, dermatofibrosarcoma protuberans; DMEM, Dulbecco’s modified Eagle’s medium; FBS, fetal bovine serum; SF, serum-free

Next, we tested if the presence of ADSCs in the basal compartment could induce a stronger response than that generated by ADSC-CM in a Transwell system (Fig. 5B). As shown in Fig. 5A, the effect of ADSCs on DFSP-migrated cell counts was tested using the Transwell system, in which DFSP cells were incubated with ADSCs (experimental), DMEM/F12+10% FBS (positive control), or DMEM/F12 SF (negative control). At 12 h, 4.7±1.2 DFSP cells had migrated in the negative control group, whereas 52.5±2.4 cells had migrated in the experimental group (*p*<0.05, Fig. 5C). At 24 h, 6.2±2.2 cells showed migration in the negative control group, whereas the number was 38.2±12.6 cells in the experimental group (*p*<0.05, Fig. 5D). The results showed that treatment with ADSCs more significantly promoted the migration of DFSP cells at 12 and 24 h time points (11.17- and 6.16-fold, respectively) compared with that observed in negative control conditions (DMEM/F12 SF).

### ADSC-CM enhanced DFSP cell invasion

**Fig. 6:**
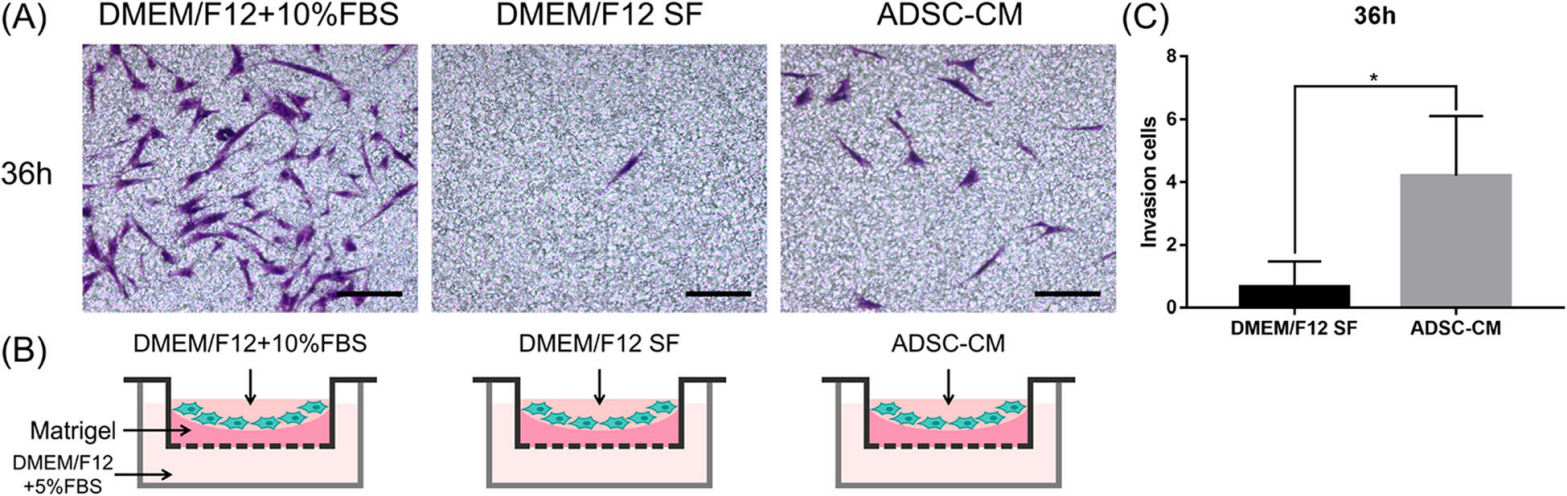
Effect of ADSC-CM on DFSP cell invasion by a Transwell assay with pre-coated Matrigel. **A** The invasive DFSP cells were visualized by staining with crystal violet. Scale bars=50 μm. **B** Illustration of cell invasion assay using Transwell with pre-coated Matrigel in different conditions. DFSP cells were seeded onto the upper chambers with DMEM/F12+10%FBS (positive control), DMEM/F12 SF (negative control), or ADSC-CM (experimental), and DMEM/F12+5%FBS was added to the lower chambers. Cell invasion was then determined at 36 h. **C** The number of invasive cells at 36 h. Invasive cells were counted in five random low-power fields per chamber using an inverted microscope. ^*^p<0.05 indicates significant differences between the experimental and negative control groups determined in three independent experiments. ADSCs, adipose tissue-derived stem cells; CM, conditioned medium; DFSP, dermatofibrosarcoma protuberans; DMEM, Dulbecco’s modified Eagle’s medium; FBS, fetal bovine serum; SF, serum-free

The invasive properties of DFSP cells allow them to digest the Matrigel matrix, a basement membrane preparation extracted from the Engelbreth-Holm-Swarm mouse sarcoma. As shown in Fig. 6A, the effect of ADSC-CM on DFSP-invaded cell counts was tested using a Transwell system with pre-coated Matrigel; DFSP cells were treated with DMEM/F12+10%FBS (positive control), DMEM/F12 SF (negative control), or ADSC-CM (experimental). At 36 h, 0.7±0.8 cells in the negative control group had invaded the lower surface, whereas in the experimental group, 4.2±1.9 cells showed invasion (*p*<0.05, Fig. 6C). The results showed that treatment with ADSC-CM could promote the invasion of DFSP cells at 36 h (7-fold) more significantly than the negative control could (DMEM/F12 SF).

### Co-cultured DFSP/ADSC-CM enhanced angiogenic properties in vitro

**Fig. 7:**
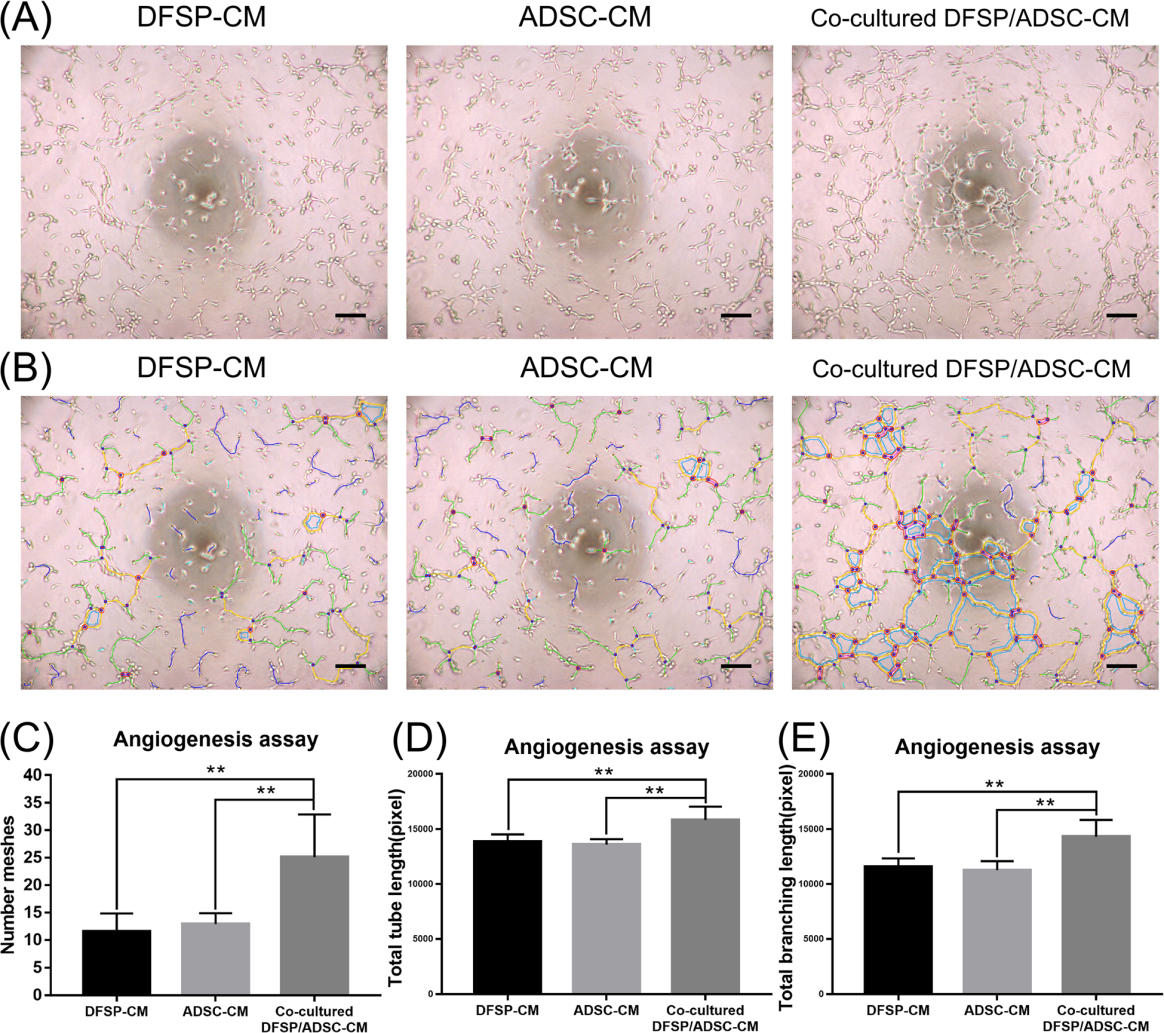
Induction of angiogenesis. **A** Representative images of HUVECs forming tubes upon treatment with different CMs. Scale bars=500 μm. **B** Representative images of HUVECs forming tubes upon treatment with different CMs with auxiliary lines. Scale bars=500 μm. Nodes: Red (#ff1303), Junction: Blue (#0035ff), Master Junction: OrangeRed (#ff2d00), Mesh: Deepskyblue (#06adfd), Segment: Magenta (#f00efc), Master Segment: Gold (#ffd300), Branch: LimeGreen (#23e500), Twig: Cyan (#19e7fb), Isolated twig: Aqua (#00f7fa), Extremity: Crimson (#f70133), Extremity edge: Yellow (#f7eb00), Isolated element: Mediumblue (#0005fe). **C** Number of meshes, **D** total tube length, and **E** total branch length of HUVECs induced by ADSC-CM, DFSP-CM, and co-cultured DFSP/ADSC-CM at 4 h, respectively. ^**^p<0.01 indicates significant differences between the co-cultured DFSP/ADSC-CM and control groups (ADSC-CM or DFSP-CM) in at least three independent experiments. ADSCs, adipose tissue-derived stem cells; CM, conditioned medium; DFSP, dermatofibrosarcoma protuberans; DMEM, Dulbecco’s modified Eagle’s medium; HUVECs, human umbilical vein endothelial cells

To evaluate the effect of proteins secreted by ADSCs, DFSP cells, and co-cultured DFSP cells and ADSCs on angiogenesis, HUVECs were incubated with different CMs (ADSC-CM, DFSP-CM, and co-cultured DFSP/ADSC-CM) that led to the formation of tubular networks. These were visible through inspection under an inverted light microscope after 4 h of incubation (Fig. 7A, B). We observed a significant increase in the number of meshes, total tube length, and total branch length of tubular networks formed by HUVECs cultured with co-cultured DFSP/ADSC-CM compared with those observed in the control groups (ADSC-CM or DFSP-CM; *p*<0.05, Fig. 7C–E).

### ADSCs increased the expression of DFSP-related genes and the protein levels

**Fig. 8:**
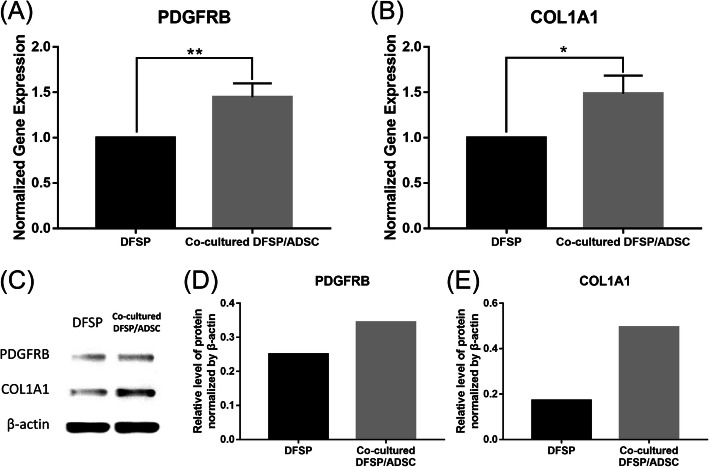
Effect of ADSCs on DFSP-related gene and protein expression in DFSP cells. **A** Beta-type platelet-derived growth factor receptor (PDGFRB) and **B** collagen type I alpha 1 chain (COL1A1) gene expression levels in DFSP cells in a mono-culture (control group) or a co-culture with ADSCs (experimental group). Data were normalized to the level of the control group. Each group was tested in triplicate. Results are shown as means±standard deviations. ^*^p<0.05 and ^**^p<0.01 indicate significant differences in relation to the control group. The protein levels of PDGFRB and COL1A1 (**C**-**E**) were measured by Western blotting in DFSP cells in the control and experimental groups. ADSCs, adipose tissue-derived stem cells; DFSP, dermatofibrosarcoma protuberans

After DFSP cells were cultured with ADSCs using the Transwell system for 24 h, the mRNA levels of PDGFRB and COL1A1 in DFSP cells showed 1.4-fold and 1.5-fold increases, respectively, compared with the levels in mono-cultured DFSP cells (*p*<0.05, Fig. 8A, B). After 48 h, in the co-cultured cells, the protein levels of PDGFRB were increased moderately and the protein levels of COL1A1 were increased significantly compared with those in the mono-cultured DFSP cells (Fig. 8C–E). Similar trends were observed in the qRT-PCR and Western blotting experiments.

### ADSCs increase growth factor gene expression in DFSP cells and growth factor secretion in the co-cultured DFSP microenvironment

**Fig. 9:**
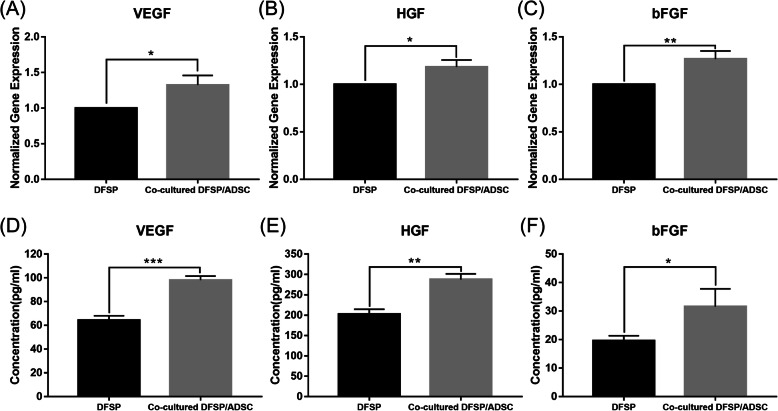
Effect of ADSCs on growth factor gene expression and protein secretion in DFSP cells and microenvironment. **A** Vascular endothelial growth factor (VEGF), **B** hepatocyte growth factor (HGF), and **C** basic fibroblast growth factor (bFGF) gene expression levels in DFSP cells in a mono-culture (control group) or co-culture with ADSCs (experimental group). Data are normalized to the level of the control group. Levels of **D** VEGF, **E** HGF, and **F** bFGF secretion in the supernatants of DFSP-mono-culture (control group) and DFSP-ADSC-co-culture (experimental group) at 48 h were detected by ELISA. ^*^p<0.05, ^**^p<0.01, and ^***^p<0.001 indicate significant differences in relation to the control group in three independent experiments. ADSCs, adipose tissue-derived stem cells; DFSP, dermatofibrosarcoma protuberans; ELISA, enzyme-linked immunosorbent assay

After DFSP cells were cultured with ADSCs using the Transwell system for 24 h, the mRNA expression of the proangiogenic genes *VEGF*, *HGF*, and *bFGF* in DFSP cells was elevated 1.32-fold, 1.2-fold, and 1.4-fold, respectively, compared with those in the mono-cultured DFSP cells (*p*<0.05, Fig. 9A–C). After 48 h, the levels of VEGF, HGF, and bFGF secretion in the collected supernatants from the co-culture (experimental group) were significantly higher compared with those in the supernatants of the mono-cultured DFSP cells (control group). The concentration of VEGF in the experimental group was 98.0±3.5 pg/mL, whereas it was 64.2±3.9 pg/mL in the control group (*p*<0.0001, Fig. 9D). The concentration of HGF in the experimental group was 287.4±14.1 pg/mL, whereas it was 202.5±12.0 pg/mL in the control group (*p*<0.01, Fig. 9E). The concentration of bFGF in the experimental group was 31.6±6.3 pg/mL, whereas it was 19.6±1.8 pg/mL in the control group (*p*<0.05, Fig. 9F).

## Discussion

The regenerative therapeutic efficacy of cell-assisted lipotransfer is dependent on the presence of ADSCs, although the stromal microenvironment and hormonal secretions of the adipose tissue are possibly involved in tumor progression [37]. There are only a few studies assessing the oncological outcomes of the interaction between ADSCs and malignant neoplasms of mesenchymal origin [17]. None of these studies have guided the therapeutic application of ADSCs in DFSP.

This is the first study to demonstrate the effects of human ADSCs on human DFSP cells by co-culture in vitro; we found that ADSCs promoted the proliferation, migration, invasion, and angiogenic properties of DFSP cells. Furthermore, in DFSP cells, changes in *PDGFRB* and *COL1A1* gene expression and protein levels were observed. Additionally, a remarkable change in the secreted protein levels of *VEGF, bFGF*, and *HGF* in the co-cultured microenvironment was observed, which plays an important role in mediating the tumor-promoting effect. These changes strongly point toward serious adverse biological consequences that may arise in the in vivo co-presence of ADSCs and DFSP cells.

Our results clearly show an increased proliferation rate in DFSP cells after co-culture with ADSC-CM. These changes suggest that direct physical contact between ADSCs and DFSP cells is not required for ADSCs to regulate the proliferation of DFSP cells, and the major mechanism underlying this may be related to their paracrine activity in the microenvironment. In sarcoma, a recent report has also shown that ADSCs trigger osteosarcoma cell proliferation in vitro, and these results were both observed in the co-culture and CM groups [27]. Meanwhile, ADSCs facilitate cell proliferation in solid malignancies of non-mesenchymal origin, including tumors of the endometrium, breast, ovary, gastric area, lung, melanoma, pancreas, and thyroid [3]. However, exposure of glioma cells to CM for 24 h and 48 h did not alter cell proliferation [25]. Furthermore, previous studies have reported that ADSCs inhibit prostate cancer cell proliferation-inducing apoptosis [38], hepatocellular carcinoma cell proliferation and division [39], and pancreatic ductal adenocarcinoma cell proliferation by altering cell cycle progression [40]. Therefore, until recently, there is no consensus in the literature regarding the effect of ADSCs on tumor cell proliferation due to the differences in cell source of cancer, model of cancer tested, or species studied [25].

Next, we observed that ADSC-CM and ADSCs could boost the migration of DFSP cells through indirect co-culture. These results are consistent with those of previous reports on the increased migration of skin tumor cells, such as malignant melanoma cells [26], squamous cell carcinoma cells [12], and non-skin tumor cells, such as breast cancer cells (line MCF7) [41] and gastric cancer cells [42]. Even in the absence of the physical presence of ADSCs, the secretome of ADSCs can promote DFSP cell migration. Interestingly, this effect was more prominent in the co-culture Transwell system, where DFSP cells shared the same medium with ADSCs without direct physical contact. This can be explained by the fact that the crosstalk between different cells together in the same microenvironment could enhance action [43].

Increased migration, a key in the process of cellular invasiveness, is involved in the degradation of the basement membrane and extracellular matrix [44]. The cell invasion assay demonstrated that ADSC-CM could stimulate DFSP cells to degrade the matrix promptly and to invade through the basement membrane. In other words, the invasiveness of DFSP cells was increased. This is consistent with the findings of previous studies on the effect of ADSCs on the metastatic ability of cancer cells, including all tumors of mesenchymal origin [27] and non-mesenchymal origin [3].

Subsequently, the co-cultured DFSP/ADSC-CM facilitated the formation of more tubular networks in HUVECs than did ADSC-CM or DFSP-CM alone. This suggests that the interaction between DFSP cells and ADSCs can regulate paracrine signaling in the TME to enhance angiogenesis, which is essential in the pathogenesis of rapid growth and metastasis in solid tumors [45]. Clearly, ADSCs have powerful angiogenic and vasculogenic capabilities in the development and progression of a wide variety of cancers, including malignant melanoma cells [26], squamous cell carcinoma cells [12], breast cancer cells [46], lung adenocarcinoma cells [47], and glioblastoma [48].

Finally, to confirm the possible mechanisms by which ADSCs enhance the malignant properties of DFSP cells, the main features of DFSP in the co-cultured microenvironment were evaluated. The expression of PDGFRB and COL1A1 in DFSP cells co-cultured with ADSCs was found to be higher at the mRNA and protein levels compared with those in controls, which was not surprising.

Soft tissue sarcomas have been suggested to contain PDGF autocrine loops. Co-expression of ligands and receptors has been observed in clinical samples of fibroblast-derived tumors, such as DFSP [49]. DFSP presents with specific cytogenetic features, such as reciprocal translocations t(17;22) (q22;q13.1) or supernumerary ring chromosomes derived from t(17;22) [50, 51]. The result of this rearrangement is the upregulation of COL1A1-PDGFB fusion proteins that are processed to form mature PDGFB and then to activate PDGFRB [52] to form an autocrine loop [53], rendering tumor cell proliferation and survival dependent on PDGFRB signaling [54]. Notably, PDGFB confers a tumorigenic phenotype to human tumor cells bearing PDGFBR but not to cells devoid of receptors [55]. As is well-known, PDGFB can act on a variety of cells by stimulating mitogenicity and chemotaxis [56]. PDGFB can also upregulate the expression of its own receptor (PDGFRB) on capillary endothelial cells to stimulate angiogenesis [57] through its ability to recruit pericytes and to improve the development of vascular smooth muscle cells [58].

Considering this, based on the high expression of PDGFRB in DFSP cells, ADSCs further promoted DFSP cells to express higher levels of PDGFRB, which amplified the effectiveness of PDGFRB signaling. These findings were consistent with the findings observed in breast cancer cells reported in the literature, with data suggesting that tumor cell-derived PDGFB/PDGFRB signaling pathway is an important factor in governing the microenvironment interaction between tumor cells and local tissue-resident stem cells [46].

Furthermore, in DFSP, the processing of the chimeric COL1A1-PDGFB protein into PDGFB dimers also results in the production of significant quantities of the COL1A1 chains. These would be combined as trimers with COL1A2 chains and processed into mature collagen fibers in the extracellular medium [59]. Through collagen bundles, the neoplasm can invade laterally and deeply along the connective tissue septae to proliferate [15]. Thus, ADSCs could promote DFSP migration and invasion via further increasing the COL1A1 expression level in DFSP cells. Recent studies have also found that COL1A1 appears to exert an oncogenic effect, which promotes tumor migration by rearranging the actin cytoskeleton and regulating the planar polarity of the cells [60, 61]. Moreover, increased COL1A1 levels were associated with poor survival [62].

Interestingly, according to studies, in keloid, one of the benign skin tumors [63], ADSCs could reduce the expression of COL1A1 in keloid fibroblasts and deposition of collagen in keloid tissue ex vivo. Currently, the reasons and mechanisms for these differential impacts of ADSCs on benign and malignant tumors are unclear. Thus, further studies should be conducted.

Meanwhile, more proangiogenic factors such as VEGF, HGF, and bFGF were detected in co-cultured DFSP/ADSC supernatant than in mono-cultured DFSP-supernatant. Furthermore, the expression of VEGF, HGF, and bFGF in DFSP cells co-cultured with ADSCs was found to be increased. In other words, ADSCs not only autocrined VEGF, HGF, and bFGF but also promoted the expression of VEGF, HGF, and bFGF in DFSP cells. This suggests that angiogenic efficiency was more greatly enhanced in co-culture TME. These findings are consistent with those of the previous literature [3]. Within a variety of tumor types and pathways, ADSCs produce numerous growth factors, including VEGF, HGF, and bFGF [64], which lead to increased vascularization [3]. It is well known that VEGF, HGF, and bFGF are potent proangiogenic factors, which all have been independently implicated in angiogenesis [65–67]. Meanwhile, VEGF and bFGF have a potent synergistic effect on the induction of angiogenesis in vitro [68]. HGF has been shown to increase the expression of VEGF to initiate angiogenesis [69] and to act in synergy with VEGF to amplify angiogenesis [70, 71]. Studies have also shown that ADSCs in the breast tissue can promote invasion of breast cancer cells via a VEGF-A-dependent manner [72], and ADSCs may favor breast cancer recurrence via HGF/c-Met signaling [73].

Taken together, the co-culture of ADSCs and DFSP cells led to considerable enhancement in malignant properties of DFSP cells in vitro. This points to a potentially increased oncological risk in vivo, which should not be neglected when considering the clinical use of fat, stem cell-enriched, and isolated ADSCs in patients with DFSP or residual DFSP cells.

## Conclusion

In the current study, we first explored the interactions between ADSCs and DFSP cells using an in vitro co-culture model to understand the effects of ADSCs on tumor development. This report provides evidence that ADSCs significantly affect multiple malignant features, such as gene expression, protein secretion, proliferation, migration, invasion, and angiogenesis, of DFSP cells in vitro. Therefore, ADSCs may strongly increase the risk of DFSP tumor development in vivo if administered near malignant tumor cells. Our results need to be considered when discussing the safety of ADSC-based therapies for patients with DFSP or residual DFSP cells. The informed consent forms for such procedures should mention the increased risk of cancer and relapse and the possibility of faster growth and dissemination of a pre-existing cancer [74]. Additionally, it appears crucial to rigorously screen all patients before the injection of adipose derivatives such as fat, stromal vascular fraction, or isolated ADSCs in adjacent tissues to avoid potential co-localization of ADSCs and DFSP cells.

## Data Availability

All data generated or analyzed during this study are included in this published article and its supplementary information files.

## Abbreviations

ADSCs: Adipose tissue-derived stem cells
bFGF: Basic fibroblast growth factor
CAFs: Cancer-associated fibroblasts
CCK-8: Cell Counting Kit-8
CM: Conditioned medium
COL1A1: Collagen type I alpha 1 chain
DFSP: Dermatofibrosarcoma protuberans
DMEM: Dulbecco’s modified Eagle’s medium
ELISA: Enzyme-linked immunosorbent assay
FBS: Fetal bovine serum
HGF: Hepatocyte growth factor
hMSCs: Human mesenchymal stem/stromal cells
HUVECs: Human umbilical vein endothelial cells
MMPs: Matrix metalloproteinases
PBS: Phosphate-buffered saline
PDGFRB: Beta-type platelet-derived growth factor receptor
qRT-PCR: Real-time quantitative real-time polymerase chain reaction
SF: Serum-free
TME: Tumor microenvironment
VEGF: Vascular endothelial growth factor
